# Biocompatibility of Gd-Loaded Chitosan-Hyaluronic Acid Nanogels as Contrast Agents for Magnetic Resonance Cancer Imaging

**DOI:** 10.3390/nano8040201

**Published:** 2018-03-28

**Authors:** Cecilia Virginia Gheran, Guillaume Rigaux, Maité Callewaert, Alexandre Berquand, Michael Molinari, Françoise Chuburu, Sorina Nicoleta Voicu, Anca Dinischiotu

**Affiliations:** 1 Faculty of Biology, Department of Biochemistry and Molecular Biology, University of Bucharest, 050095 Bucharest, Romania; virginia.gheran@bio.unibuc.ro (C.V.G.); anca.dinischiotu@bio.unibuc.ro (A.D.); 2 Institut de Chimie Moléculaire de Reims, CNRS UMR 7312, Université de Reims Champagne-Ardenne URCA, F-51685 Reims CEDEX 2, France; guillaume.rigaux@univ-reims.fr (G.R.); maite.callewaert@univ-reims.fr (M.C.); francoise.chuburu@univ-reims.fr (F.C.); 3 Laboratoire de Recherche en Nanosciences—EA 4682, Plate-forme Nano’Mat, Université de Reims Champagne-Ardenne URCA, F-51685 Reims CEDEX 2, France; alexandre.berquand@univ-reims.fr (A.B.); michael.molinari@univ-reims.fr (M.M.); 4 Faculty of Pharmacy, Department of Pharmacy, Titu Maiorescu University, 004051 Bucharest, Romania

**Keywords:** gadolinium, nanogels, SVEC4-10 cell line, cytotoxicity, oxidative stress, genotoxicity

## Abstract

Although the research on nanogels incorporating Gd chelates for theranostic applications has grown exponentially in recent years, knowledge about their biocompatibility is limited. We compared the biocompatibility of Gd-loaded hyaluronic acid-chitosan-based nanogels (GdCA⊂CS-TPP/HA) with two chitosan concentrations (2.5 and 1.5 mg·mL^−1^ respectively) using SVEC4-10 murine lymph node endothelial cells. The sulforhodamine B method and released lactate dehydrogenase (LDH) activity were used as cell viability tests. Reactive oxygen species (ROS), reduced glutathione (GSH) and malondialdehyde (MDA) were measured by spectrophotometric and fluorimetric methods. Nrf-2 protein expression was evaluated by Western blot analysis and genotoxicity by alkaline comet assay. After 24 h, the cells viability was not affected by all types and doses of nanogels. The increase of ROS induced a low decrease of GSH concentration and a time-dependent raise of MDA one was produced by citric GdDOTA⊂CS-TPP/HA with a chitosan concentration of 1.5 mg·mL^−1^, at the highest dose applied. None of the tested nanogels induced changes in Nrf-2 protein expression. A slight but significant genotoxic effect was caused only by citric GdDOTA⊂CS-TPP/HA where CS concentration was 1.5 mg·mL^−1^. Our results showed a better biocompatibility with lymph node endothelial cells for Gd-loaded hyaluronic acid-chitosan based nanogels with a concentration in chitosan of 2.5 mg·mL^−1^.

## 1. Introduction

Magnetic resonance imaging (MRI) is a powerful non-invasive and non-ionizing tool for imaging tissues as well as tumours in humans and animals. It offers a high 3D spatial resolution and infinite tissue penetration [1] but suffers from poor sensitivity. In this context, complexes of paramagnetic metal ions, mostly lanthanides [2], iron oxide nanoparticles [3] as well as organic radical contrast agents [4] are used to improve the image contrast. Gadolinium-based contrast agents (GdCAs) are the most commonly used agents in clinical MRI [5] and have been used to enhance MRI sensitivity by enhancing the signal contrast between normal and tumour tissues [6,7]. To have a positive impact on the contrast, a high GdCA concentration must be injected, which—in some cases of kidney failure for example—may be problematic [8,9,10]. To overcome this drawback varied nanoplatforms integrating gadolinium chelates including lipid-based [11], polymeric [12] and silica NPs [13], as well as micelles [14], dendrimers [15] and nanogels [16] were synthesized. However, the biocompatibility and ultimately biodegradability of gadolinium-based imaging agents are crucial issues to consider since they are designed for in vivo administration [17]. For these reasons and due to their specific properties, such as hydrophilicity, biodegradability and biological activity, polysaccharides such as chitosan (CS) and hyaluronic acid (HA) are of great interest to design biocompatible polymeric nanoparticles [18,19].

CS is currently used in biomedical field due to its biocompatibility and low-toxicity [20,21]. It is a linear positively charged polysaccharide composed of N-acetyl-D-glucosamine and D-glucosamine residues and obtained by partial deacetylation of chitin [22,23]. Among all other natural polymers, CS is the only one that has a cationic character. This behaviour is based on its primary amino groups which are ionized in weakly acidic media, allowing the biopolymer to adhere to the negatively charged surfaces and thus to the cell membrane [24]. The cationic character of chitosan is responsible for its special characteristics such as muco-adhesiveness [25], antitumor [26,27], antimicrobial [28], antioxidant activities [29] and ability to interfere with cellular tight junctions [30]. Consequently, it is used in the development of new drug delivery systems [12,25,31,32,33,34,35,36,37,38].

Hyaluronic acid (HA) is a linear anionic polymer, a free un-branched glycosaminoglycan constituted of D-glucuronic acid and *N*-acetyl-d-glucosamine residues. HA is naturally occurring mainly in the extracellular matrix and connective tissues of vertebrates [39]. Because of its cell surface receptors (CD44, RHAMM, HARE and LYVE-1) it is involved in several biological processes such as endocytosis, homeostasis and intracellular signalling transduction [40,41,42]. It is biodegradable, biocompatible, bioactive, non-inflammatory and non-cytotoxic [43] and due to these advantages, it is a widely investigated biomaterial in drug delivery [44,45], tissue engineering [42,46] and molecular imaging [47,48].

The applicability of CS is hindered by its solubility, which can be increased by modifications made to the free amino groups along the chitosan backbone under mild conditions. However, in the presence of specific polyanions, chitosan has the ability to form a novel class of hydrophilic biomaterial, called hydrogels.

Many studies were focused on synthesis of hydrogels by ionic gelation using HA [49,50,51,52,53,54,55,56,57,58,59,60]. Recently, hyaluronic acid-chitosan (HA-CS) based nanogels have become a promising strategy for confinement of GdCAs in designing of new MRI probes. [60,61,62,63,64].

Up to now, only a few studies regarding the biocompatibility of these nanoparticles have been reported. Zhang et al. have shown both the safety of gadolinium-loaded CS nanoparticles against B16 cells [65] and HA-CS nanoparticles against B16, HepG2 and A549 cells [64]. HA modified CS-gadolinium nanospheres generated a low cellular cytotoxicity towards HCT 116 cells [60]. Moon et al. evaluated the toxicity profile of HA-DTPA-Gd in two cell lines (NIH3T3 and FL83B) [47] and showed that compared to NIH3T3 cells, cytoxicity in FL83B was increased at higher dose of HA-DTPA-Gd applied. Previous studies also indicated that gadolinium complexes such as GdDOTA and GdDOTP encapsulated in a hydrophilic matrix constituted of CS and HA had no impact on C6 glioma cells and fibroblasts from rat skin viabilities [61,63], that suggested a high biocompatibility with these cell lines. Nevertheless, our group demonstrated that these nanogels could induce a slight oxidative stress towards A20 lymphocytes [66]. Since these nanogels are designed for lymphatic system imaging it is important to evaluate their in vitro effects towards dedicated cell lines, such as SVEC4-10 murine lymph node cells. A preliminary study conducted with this cell line [67] seemed to indicate that under certain conditions, results similar to those demonstrated for A20 lymphocytes were obtained. To have a better insight of the biological effects induced by Gd nanogels on SVEC4-10 murine lymph node cells, the impact towards these cells of CS-HA nanogels synthesized with two CS concentrations (1.5 and 2.5 mg·mL^−1^) and two paramagnetic Gd chelates (GdDOTA and GdDOTP) was evaluated. In this respect, total cellular biomass, lactate dehydrogenase (LDH) activity, reactive oxygen species (ROS), lipid peroxidation, reduced glutathione (GSH), as well as the level of nuclear factor (erythroid-derived 2)-like 2 (Nrf-2) protein expression and the genotoxic effect by alkaline comet assay were analysed.

## 2. Materials and Methods 

### 2.1. Reagents

Sterile water was purchased from Laboratoire Aguettant, Lyon, France and sodium tripolyphosphate (TPP) from Acros Organics. DOTA and DOTP were purchased from Macrocyclics (Dallas, TX, USA). Chitosan (CS, low viscosity from shrimp shells) and sodium hyaluronate were provided by Sigma-Aldrich (St. Louis MO, USA) and bovine serum albumin by Sigma (Auckland, New Zealand). Fetal bovine serum, Dulbecco’s Modified Eagle Medium (DMEM), phosphate-buffered saline (PBS) were purchased from Gibco (Grand Island, NY, USA) and WesternBreeze^®^ Chromogenic Kits were procured from Invitrogen by Thermo Fisher Scientific Inc. (Waltham, MA, USA). In Vitro Toxicology Assay Kit Sulforhodamine B based, In Vitro Toxicology Assay Kit Lactic Dehydrogenase based, reduced glutathione (GSH), 5,5-dithio-bis-(2-nitrobenzoic acid), thiobarbituric acid (TBA); penicillin-streptomycin-amphotericin antibiotic mixture, 2′,7′-dichlorofluorescein diacetate (DCFH-DA) and 1,1,3,3-tetramethoxypropane were provided by Sigma (St. Louis, MO, USA). SVEC4-10 endothelial cell line was obtained from American Type Culture Collection (ATCC), Rockville, MD, USA. OxiSelect™ Comet Assay Kit (3-Well Slides) was purchased from Cell Biolabs, specific primary antibodies Nrf-2 from Santa Cruz Biotechnology, Inc. (Heidelberg, Germany) and all other chemicals used were of high purity and available from commercial suppliers.

### 2.2. Synthesis and Characterization of Nanogels (NGs)

Four stock solutions of chitosan were prepared by dissolution of the CS powder at concentrations of 2.5 mg·mL^−1^ or 1.5 mg·mL^−1^ in a 10% (*m*/*v*) citric acid or acetic acid aqueous solution and stirred overnight.

Nanogels were obtained by an ionotropic gelation process [61,63]. The polyanionic phase, that is, HA (0.8 mg·mL^−1^) and TPP (1.2 mg·mL^−1^) in water (4.5 mL), were added dropwise to the CS solution (9 mL) under sonication (750W, amplitude 32%) to obtain stable nanosuspensions. The gadolinium complex (GdDOTP or GdDOTA) was previously dissolved in the polyanion solution. At the end of the addition, magnetic stirring was maintained for 10 min. Unloaded nanogels were obtained in the same way, omitting the gadolinium complex. Nanosuspensions were then freeze-dried, using glucose 15% (*m*/*v*) as a cryoprotectant. Nanoparticle average hydrodynamic diameters (D_H_) and polydispersity indexes (PdI) were determined by Dynamic Light Scattering (DLS) (Malvern Zetasizer Nano-ZS, Malvern Instruments, Worcestershire, UK). Each nanosuspension was analysed in triplicate at 20 °C at a scattering angle of 173°, for each sample, after 1/20 dilution in water. Pure water was used as a reference dispersing medium. ζ-(zeta) potential data were collected through electrophoretic light scattering at 20 °C, 150 V, in triplicate for each sample, after 1/20 dilution in water. The instrument was calibrated with a Malvern—68 mV standard before each analysis cycle.

The shape and the surface morphology of the nanogels were investigated by atomic force microscopy (AFM) (Catalyst, BrukerNano) in tapping mode. Samples were prepared by placing a drop of nanoparticle suspension on a freshly-cleaved mica sheet and the experiments were performed in fluid tapping mode to keep the integrity of the NPs. AFM images were generated with a scan rate of 1 Hz and 512 lines per image. Experiments were performed at constant room temperature. During the scans, proportional and integral gains were increased to the value just below the feedback started to oscillate. Images were processed only by flattening to remove background slopes.

Gadolinium nanogel loading was determined on nanoparticle suspensions by ICP-OES. The nanoparticle suspension was incubated overnight in a 1:3 (*v*/*v*) mixture of HCl (37%) and HNO_3_ (69%). Volumetric dilutions were carried out to achieve an appropriate Gd concentration within the working range of the method. Samples were analysed using Thermo Scientific iCAP 6300 series Duo ICP spectrometer. Gd emission intensity was correlated to Gd concentration by means of a calibration curve which was previously established from Gd(NO_3_)_3_ ICP-OES standard. Solutions used for the calibration were obtained by dilution of increasing amounts of Gd(NO_3_)_3_ standard with unloaded nanogels previously incubated under acidic conditions, as described above.

### 2.3. Cell Culture Conditions and Treatment

Adherent murine SVEC4-10 cells (ATCC CRL 2181) were cultured in DMEM medium supplemented with 10% heat inactivated fetal bovine serum, 1% antibiotic-antimycotic mix and maintained at 37 °C in a humidified air atmosphere with 5% CO_2_ for specific time periods. The cells were seeded at a density of 5 × 10^4^ cells/mL into 24 well plates (for Sulforhodamine B and lactate dehydrogenase), 10^5^ cells/mL in six well plates (for intracellular ROS) and in 25 cm^2^ culture flasks (for lipid peroxidation, glutathione reduced and Nrf-2 protein expression levels, as well as for comet assay). These were exposed to 1 μM, 2.5 μM, 5 μM and 10 μM of GdCA⊂CS-TPP/HA nanogels (GdCA = GdDOTA or GdDOTP) for 6 and 24 h. CS-TPP/HA nanogels were used as controls.

Before the treatment of cells, the stock solutions of nanogels with concentrations in range of 49–907 µM (presented in Table 1) were diluted in DMEM medium finally reaching the doses which were used for the cell incubations in biological experiments (1, 2.5, 5 and 10 µM Gd).

### 2.4. Preparation of Cell Lysate

After each interval, cells were harvested from culture flasks using 0.25% trypsin solution and centrifuged at 1500 rpm, 4 °C for 5 min. Then, the cells were resuspended in 0.1 mL of PBS, sonicated (three times for 30 s on ice) using an Hielscher Ultrasonic processor UP50H and centrifuged at 5000 rpm, 4 °C for 10 min. The supernatant was aliquoted and stored at −80 °C and used for subsequent biochemical determinations. Protein concentration was measured according to Bradford method [68] using bovine serum albumin (BSA) as standard protein.

### 2.5. Sulforhodamine B (SRB) Assay

The cell viability was performed using a commercial kit (In Vitro Toxicology Assay Kit, Sulforhodamine B based, St. Louis MO, USA) according to manufacturer’s instructions. This method is used for cell density determination, based on the measurement of total cellular biomass. After the treatment with GdCA⊂CS-TPP/HA nanogels (GdCA = GdDOTA or GdDOTP) the cells were fixed for one hour at 4 °C with 50% TCA solution (1/4 volume of growth medium). Afterwards, cells were stained with 0.4% sulforhodamine B for 20 min, then, rinsed quickly with 1% acetic acid. The incorporated dye was solubilized with 10 mM Tris base solution. The absorbance was measured at 550 nm using a multireader (Thermo Scientific Appliskan, Vantaa, Finland) and the results were expressed relative to control cells.

### 2.6. LDH Assay

As a parameter of cytotoxicity in terms of cell membrane damage, LDH assay using the In Vitro *Toxicology Assay Kit*, Lactic Dehydrogenase based (Sigma-Aldrich, St. Louis, MO, USA), in accordance with the manufacturer’s instructions, was performed [69]. The cells were seeded in 24 well plates and exposed at different concentrations of GdCA⊂CS-TPP/HA nanogels (GdCA = GdDOTA or GdDOTP) for 6 and 24 h. Afterwards, a volume of 50 μL of culture supernatant from each well was transferred to a new plate along with 100 μL of LDH mix solution. After 30 min of incubation at room temperature, the reaction was stopped with 1M HCl. The absorbance was measured using a microplate reader (Thermo Scientific Appliskan) at 450 nm and the results were expressed relative to unloaded nanogels.

### 2.7. Intracellular ROS Measurement

Intracellular ROS generation was measured using 2′,7′-dichlorofluorescein diacetate (DCFH-DA) (Sigma-Aldrich, St. Louis, MO, USA). Briefly, a 10 mM DCFH-DA stock solution was diluted 1000-fold in free of serum cell culture medium to yield a 10 µM working solution. After exposure of SVEC4-10 cells to 2.5 μM and 10 μM of GdCA⊂CS-TPP/HA nanogels (GdCA = GdDOTA or GdDOTP) for 6 and 24 h, these were washed with PBS and incubated in a 2 mL working solution of 10 µM DCFH-DA for 45 min at 37 °C in the dark. Then, the cells were washed with PBS and resuspended in PBS for analysis. The intensity of DCF fluorescence was measured at an excitation and emission wavelengths of 485 and 530 nm, respectively, using a FlexStation 3 Multi-Mode Microplate Reader from Molecular Devices LLC and the SoftMax Pro software. The fluorescence intensity was reported to the total number of viable cells and expressed as % of unloaded CS-TPP/HA NGs.

### 2.8. Lipid Peroxidation Measurement

Determination of lipid peroxidation, one of the markers of lipids oxidative degradation was assessed using thiobarbituric acid (TBA). Malondialdehyde (MDA) was measured according to the method described by Del Rio et al. [70]. After 6 and 24 h exposure at GdCA⊂CS-TPP/HA nanogels (GdCA = GdDOTA or GdDOTP), cells were collected and washed with PBS solution. Briefly, 200 μL of cell suspension was mixed with 700 μL of 0.1 M HCl and incubated at room temperature for 20 min. After that, 900 μL of 0.025 M thiobarbituric acid were added and the mixture was maintained for 65 min at 37 °C. The MDA-TBA adduct was quantified fluorometrically (λexc/em = 520/549 nm) using a Jasco FP-750 spectrofluorometer (Tokyo, Japan). The results were expressed as nmoles of MDA/mg of cellular protein and MDA concentration was calculated using a calibration curve with 1,1,3,3-tetramethoxy propane concentrations in the range of 0.5–5 μM.

### 2.9. GSH Assay

GSH concentrations were measured using the Glutathione Assay Kit from Sigma-Aldrich according to the manufacturer’s instructions. After treatment, cell lysates were deproteinized with an equal volume of 5% 5-sulfosalicylic acid (SSA) and centrifuged at 10,000 rpm, 4 °C for 10 min. A volume of 10 μL of each sample was placed in a 96-well microtiter plate and then, 150 μL of working mixture were added. After 5 min incubation at room temperature, the absorbance was measured spectrophotometrically at a wavelength of 412 nm and the results were expressed in nmoles GSH/mg of protein.

### 2.10. Western Blot Assay

SVEC4-10 cells were exposed to 2.5 µM and 10 µM GdCA⊂CS-TPP/HA nanogels (GdCA = GdDOTA or GdDOTP) for 6 and 24 h. After exposure, cells were collected, washed with PBS and lysed using the Hielscher Ultrasonic processor UP50H. Protein concentration was determined using Bradford reagent and serum bovine albumin as standard. The protein samples (25 µg) were resolved by 10% SDS gels at 90 V and transferred to polyvinylidene difluoride membranes (Merck KGaA, Darmstadt, Germany) at 350 mA for 90 min in a wet transfer system (Bio-Rad, Hercules, CA, USA). After blocking, membranes were incubated with dilute solution (1:250) of primary antibodies including Nrf-2 (Santa Cruz Biotechnology Inc., Heidelberg, Germany) and anti-β-actin (Sigma) overnight at 4 °C. Then, membranes were incubated with anti-rabbit secondary antibodies coupled with alkaline phosphatase at room temperature for one hour. The protein bands were detected using BCIP/NBT as chromogenic substrate and visualized with the Bio-Rad ChemiDoc Imaging System (Bio-Rad, Hercules, CA, USA). Protein expression was quantified with Bio-Rad Image Lab software (version 5.2, Bio-Rad, Hercules, CA, USA), normalized to actin and the results were represented as percentage from controls.

### 2.11. Comet Assay

The Comet Assay, also known as single cell gel electrophoresis (SCGE), is a visual and sensitive technique for measuring DNA breakage in individual mammalian cells. After cells exposure to 2.5 μM and 10 μM of GdCA⊂CS-TPP/HA (GdCA = GdDOTA or GdDOTP) NGs, DNA damage was analysed by Single cell gel electrophoresis kit (Cell Biolabs, INC). SVEC4-10 cells were harvested and re-suspended in ice-cold PBS at 1 × 10^5^ cells/mL density. A cell suspension of 8 µL was mixed with 80 µL of low melting agarose and then, 75 µL were pipetted onto a comet-slide. The slides were maintained horizontally in dark at 4 °C for 15 min, then, these were transferred in pre-chilled lysing solution at 4 °C for one hour. Afterwards, lysis solution was removed and slides were immersed in alkaline solution for 30 min at 4 °C in the dark. The slides were washed twice for 5 min with pre-chilled deionized water and then, these were electrophoresed in a horizontal chamber at low voltage (300 mA, 25 V) for 30 min. Following electrophoresis, the slides were immersed in 70% ethanol for 5 min. The samples were allowed to air dry, stained with a Vista Green DNA dye for 15 min and visualized by epifluorescence microscopy (Olympus IX 71, Tokyo, Japan) using a fluorescein isothiocyanate (FITC) filter. Data were analysed using the Open Comet program. The percentage of DNA in tail was selected as indicator of DNA damage.

### 2.12. Statistical Analysis 

Statistical analysis was performed using Student’s *t* test (Microsoft Excel) and all values were expressed as average ± SD (*n* = 3). A *p* value < 0.05 was considered statistically significant and data were represented as graphics relative to control which was considered 100%. Each test was performed in three independent experiments.

## 3. Results

### 3.1. Physico-Chemical Characteristics of GdDOTA and GdDOTP Based Nanogels

GdCA⊂CS-TPP/HA nanogels (GdCA = GdDOTA or GdDOTP) were prepared by a one-step ionotropic gelation process [61,63]. This method relied upon the establishment of multivalent electrostatic interactions between CS derivatives (polycationic) and HA (polyanionic). The resulting supramolecular network could be reinforced by cross-linking mediated by small anionic cross-linkers such as TPP. The average hydrodynamic diameters of GdCA⊂CS-TPP/HA NGs were determined by DLS as well as the polydispersity index of the NP population (Table 1). Nanoparticle zeta potential (ζ) which was indicative of their outermost surface charge was determined by ELS.

For each CS concentration, nanogel mean sizes and their zeta potential were comparable, as well as the polydispersity indexes (PdI) of the corresponding nanosuspensions. Gd loadings were superior for formulations where CS concentration was 2.5 mg·mL^−1^.

**Figure 1:**
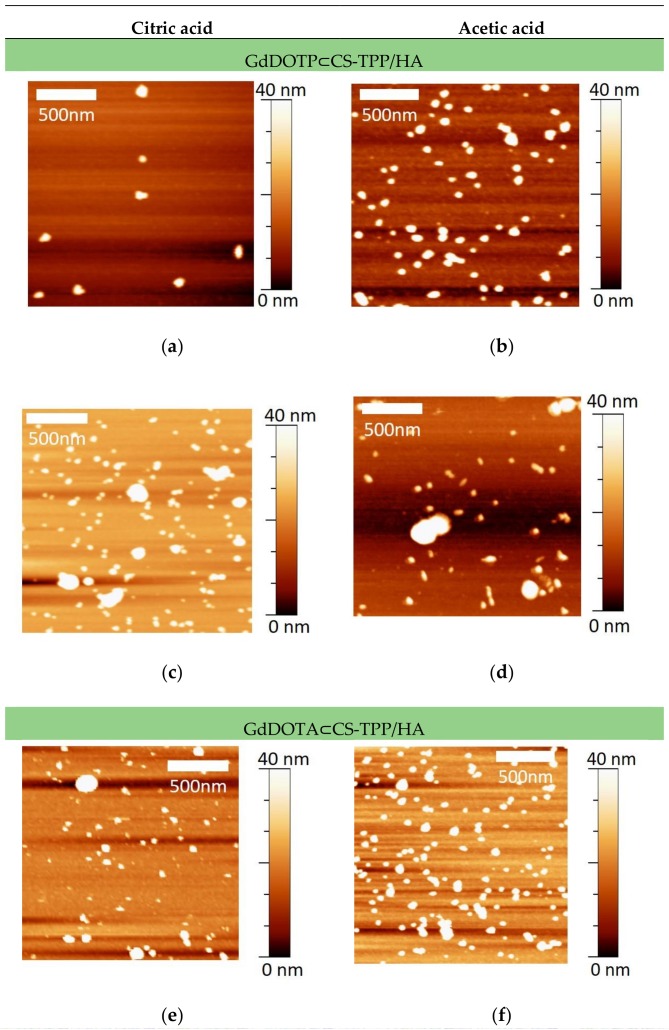
Atomic force microscopy (AFM) images in liquid of nanogels (**a**–**f**).

We have recently shown that in order to have a better insight in nanogel mean sizes, DLS data should be systematically consolidated by AFM observations in tapping mode [67]. Moreover, when AFM is performed in liquid mode, that is, in conditions close to the ones used in DLS, this technique is particularly well suited to image fragile samples such as hydrogels because friction and shear forces are negligible. By comparison to DLS, the advantage of AFM relies on the fact that it does not require the application of mathematical models to obtain size information. Therefore, AFM is more reliable to distinguish between individual nanoparticles and particle aggregates. By DLS, this is trickier and results in an overestimation of nanoparticle size [67]. AFM images were acquired in liquid mode for all the nanosuspensions (Figure 1).

No topological differences could be noticed according to CS concentration, the acid or the gadolinium contrast agent. In any case, NGs diameters calculated from the AFM images were highly inferior to 100 nm (Table 2) as already demonstrated before [66].

### 3.2. Effect of GdDOTA and GdDOTP Based Nanogels on Cell Viability 

A very important aspect in developing new nanogels for lymph node MRI is the evaluation of their toxicity when interacting with cells. In this study, the SVEC4-10 cells survival to increasing doses of GdCA⊂CS-TPP/HA NGs (GdCA = GdDOTA or GdDOTP) was tested after 6 and 24 h of exposure, using Sulforhodamine B viability test. First, we tested the viability of cells exposed to unloaded NGs compared to untreated cells. These results revealed the unloaded NGS were not toxic up to 24 h (Table 3).

**Figure 2:**
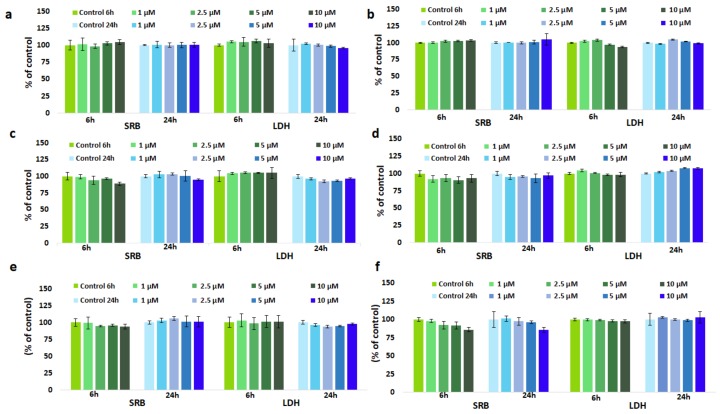
Viability and cytotoxicity evaluation in SVEC4-10 cells in the presence of increased concentrations of (**a**) GdDOTP citric acid, 2.5 mg·mL^−1^ CS; (**b**) GdDOTP acetic acid, 2.5 mg·mL^−1^ CS; (**c**) GdDOTP citric acid, 1.5 mg·mL^−1^ CS; (**d**) GdDOTP acetic acid, 1.5 mg·mL^−1^ CS; (**e**) GdDOTA citric acid, 1.5 mg·mL^−1^ CS; (**f**) GdDOTA acetic acid, 1.5 mg·mL^−1^ CS, after 6 and 24 h. Unloaded CS-TPP/HA NGs obtained in citric and acetic acid were used as controls. Data are expressed as average ± SD (*n* = 3).

Viabilities of Gd-NGs treated cells in percent of total cellular biomass of the control (cells treated with unloaded NGs) are displayed in Figure 2**.** No significant change in cell viability was registered at any concentrations applied up to 24 h. It can be also observed that cell survival profiles are independent on the CS concentration and type nature of acid used for nanogel preparation and the gadolinium complex encapsulated. Cytotoxicity evaluated by SRB survival assay suggested that nanogels in doses up to 10 µM were nontoxic and safe to this type of cell.

### 3.3. Effect of GdDOTA and GdDOTP Based Nanogels on Cell Membrane Integrity

The effects of nanogels on SVEC 4–10 cells were investigated by assessing cellular membrane integrity through the LDH release. The cytotoxicity assay showed that, after 6 and 24 h of the incubation of SVEC4-10 cells with GdCA⊂CS-TPP/HA NGs in doses ranging from 1 to 10 µM no significant changes were noticed (Figure 2).

### 3.4. Effect of GdDOTA and GdDOTP Based Nanogels on Oxidative Stress

**Figure 3:**
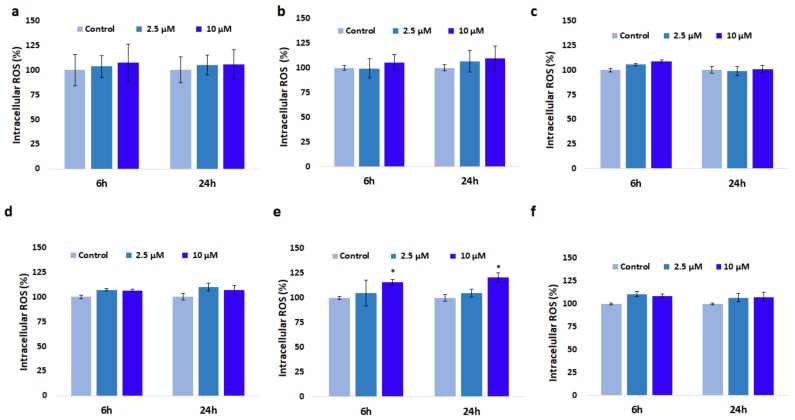
The intracellular reactive oxygen species (ROS) levels in SVEC4-10 cells in the presence of increased concentrations of (**a**) GdDOTP citric acid, 2.5 mg·mL^−1^ CS; (**b**) GdDOTP acetic acid, 2.5 mg·mL^−1^ CS; (**c**) GdDOTP citric acid, 1.5 mg·mL^−1^ CS; (**d**) GdDOTP acetic acid, 1.5 mg·mL^−1^ CS; (**e**) GdDOTA citric acid, 1.5 mg·mL^−1^ CS; (**f**) GdDOTA acetic acid, 1.5 mg·mL^−1^ CS, after 6 and 24 h Unloaded CS-TPP/HA NGs obtained in citric and acetic acid were used as controls. Data are expressed as average ± SD (*n* = 3).

A significant increase of ROS level by 15% and 20% was observed in SVEC4-10 cells exposed to 10 µM GdDOTA⊂CS-TPP/HA NGs containing 1.5 mg·mL^−1^ CS (citric acid phase) (e), after 6 and 24 h, respectively compared to control (CS-TPP/HA NGs). The fluorescence intensity of 2′,7′-dichlorodihydrofluorescein (DCF) was insignificant at the lower concentration (2.5 µM), after 6 and 24 h (Figure 3). For the rest of nanogels no major ROS generation was noticed.

### 3.5. Effect of GdDOTA and GdDOTP Based Nanogels on Lipid Peroxidation

**Figure 4:**
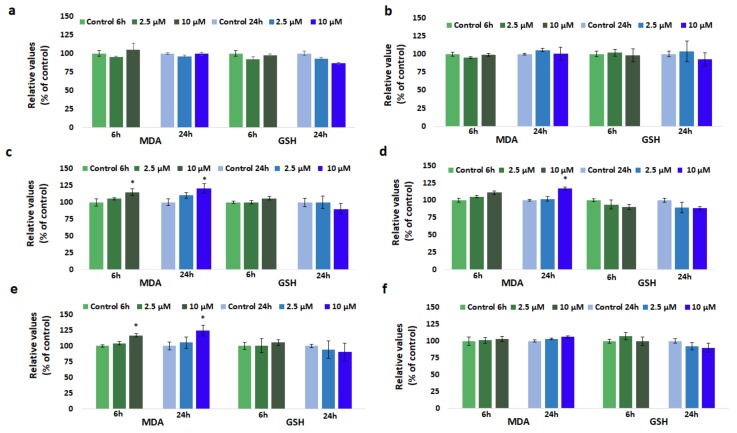
Malondialdehyde (MDA) and glutathione (GSH) relative levels in SVEC4-10 cells in the presence of increased concentrations of (**a**) GdDOTP citric acid, 2.5 mg·mL^−1^ CS; (**b**) GdDOTP acetic acid, 2.5 mg·mL^−1^ CS; (**c**) GdDOTP citric acid, 1.5 mg·mL^−1^ CS**;** (**d**) GdDOTP acetic acid, 1.5 mg·mL^−1^ CS; (**e**) GdDOTA citric acid, 1.5 mg·mL^−1^ CS; (**f**) GdDOTA acetic acid, 1.5 mg·mL^−1^ CS, after 6 and 24 h. Unloaded CS-TPP/HA NPs obtained in citric and acetic acid were used as controls. Data are expressed as average ± SD (*n* = 3).

Figure 4 shows the levels of MDA measured for SVEC4-10 cells, following their exposure to different GdCA⊂CS-TPP/HA NGs for 6 and 24 h. For endothelial cells treated with both citric and acetic GdDOTP⊂CS-TPP/HA (a and b) where CS concentration was 2.5 mg·mL^−1^, no remarkable increase of MDA level was observed. By contrast, for NGs with 1.5 mg·mL^−1^ CS, a time-dependent increase of the MDA level in endothelial cells was noticed with the most significant changes by 24%, 20% and 17% being observed after 24 h of exposure to citric GdDOTA⊂CS-TPP/HA (e) and GdDOTP⊂CS-TPP/HA (c) and acetic GdDOTP⊂CS-TPP/HA (d), respectively, as compared to unloaded ones (CS-TPP/HA NGs). It should be noticed that no modification of MDA level was observed for SVEC4-10 cells treated with acetic GdDOTA⊂CS-TPP/HA (f) nanogels.

### 3.6. Effect of GdDOTA and GdDOTP Based Nanogels on GSH Level

No time- and dose-dependent reduction of GSH level was registered in SVEC4-10 cells treated with GdDOTP⊂CS-TPP/HA (a and b) in both citric and acetic phase where CS concentration was 2.5 mg·mL^−1^ (Figure 4) but slight decreases were observed when cells were treated for 24 h with 10 µM GdCA⊂CS-TPP/HA NGs with 1.5 mg·mL^−1^ CS. In this case, GSH level was diminished by about 12% and 10% when cells were exposed to citric GdDOTA⊂CS-TPP/HA (e), GdDOTP⊂CS-TPP/HA (c) and acetic GdDOTA⊂CS-TPP/HA (f), GdDOTP⊂CS-TPP/HA (d), respectively, comparing to the corresponding controls (CS-TPP/HA NGs).

### 3.7. Effect of GdDOTA and GdDOTP Based Nanogels on Nrf-2 Protein Expression

**Figure 5:**
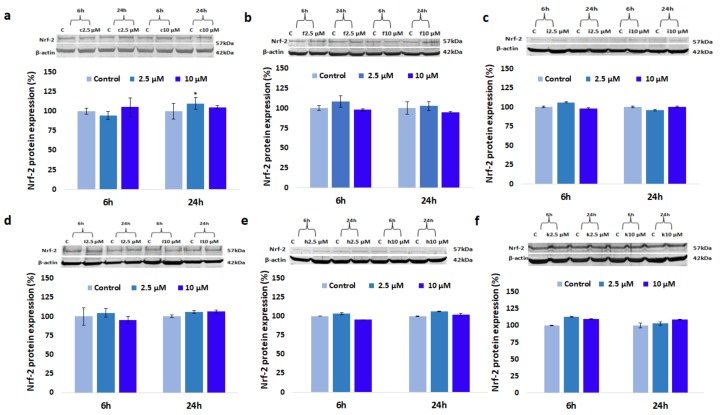
Nrf-2 protein expression in SVEC4-10 cells in the presence of increased concentrations of (**a**) GdDOTP citric acid, 2.5 mg·mL^−1^ CS; (**b**) GdDOTP acetic acid, 2.5 mg·mL^−1^ CS; (**c**) GdDOTP citric acid, 1.5 mg·mL^−1^ CS; (**d**) GdDOTP acetic acid, 1.5 mg·mL^−1^ CS; (**e**) GdDOTA citric acid, 1.5 mg·mL^−1^ CS; (**f**) GdDOTA acetic acid, 1.5 mg·mL^−1^ CS, after 6 and 24 h Unloaded CS-TPP/HA NGs obtained in citric and acetic acid were used as controls. Data are expressed as average ± SD (*n* = 3).

The expression level of Nrf-2 was measured by Western blot analysis and the results are presented in Figure 5. Insignificant changes in Nrf-2 protein level between GdCA⊂CS-TPP/HA NGs- and unloaded NGs-exposed cells were noticed during the entire analysed period. In addition, for nanogels synthesized under similar conditions (i.e., same acid and same Gd chelate) no differences were registered for both doses of CS.

### 3.8. Genotoxic Effect of GdDOTA and GdDOTP Based Nanogels

**Figure 6:**
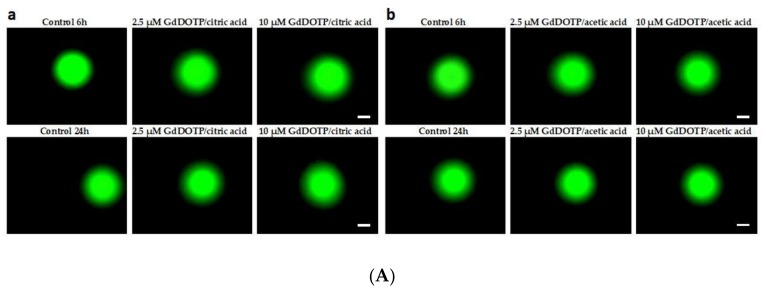

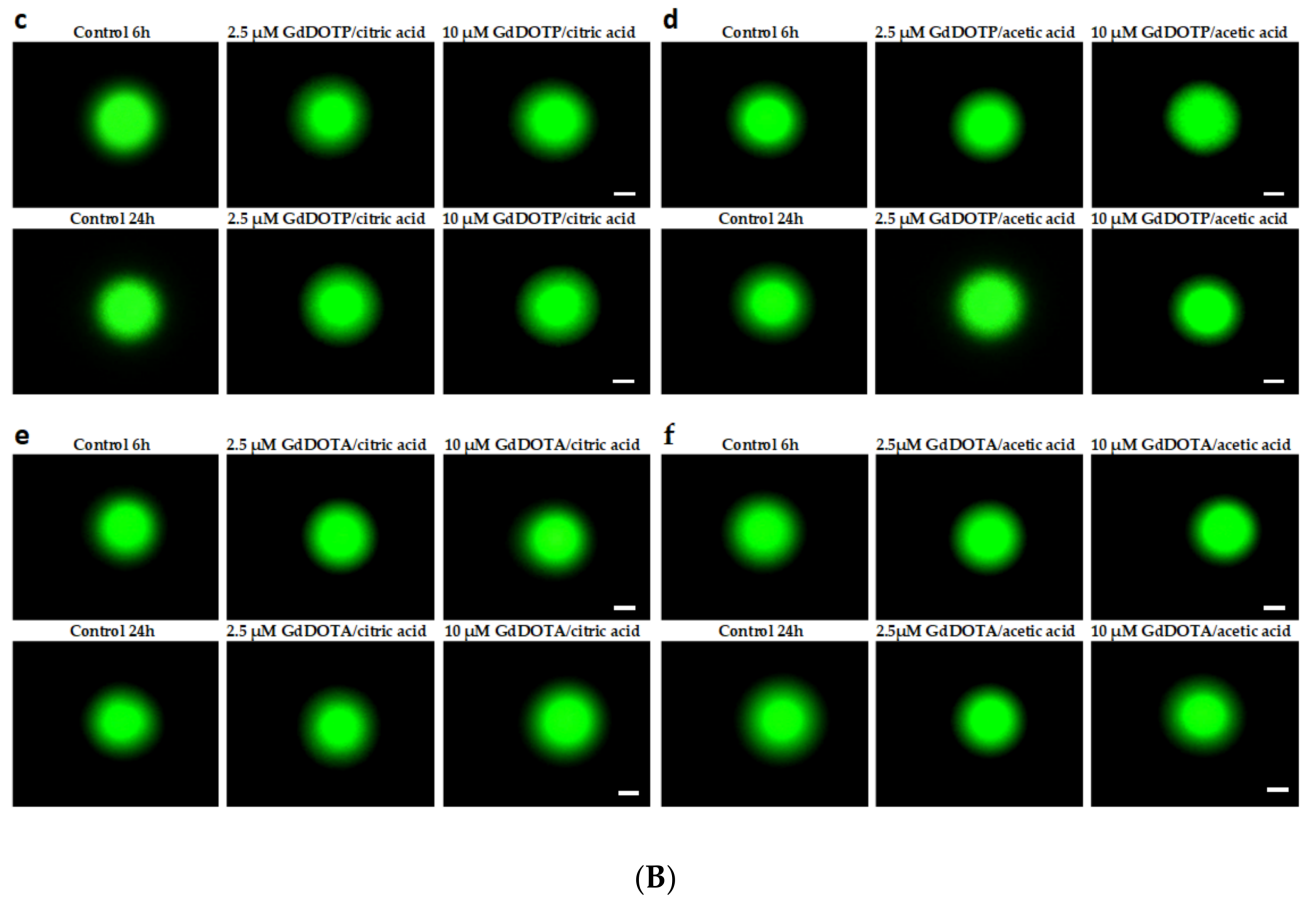
(**A**) Representative fluorescence images of SVEC4-10 cells in the presence of increased concentrations of (**a**) GdDOTP citric acid, 2.5 mg·mL^−1^ CS; (**b**) GdDOTP acetic acid, 2.5 mg·mL^−1^ CS after 6 and 24 h. Unloaded CS-TPP/HA NGs obtained in citric and acetic acid were used as controls. Scale bar = 50 µm. Magnification, ×100. The green fluorescence intensity were analysed and quantified using the OpenComet program; (**B**) Representative fluorescence images of SVEC4-10 cells, in the presence of increased concentrations of, (**c**) GdDOTP citric acid, 1.5 mg·mL^−1^ CS, (**d**) GdDOTP acetic acid, 1.5 mg·mL^−1^ CS, (**e**) GdDOTA citric acid, 1.5 mg·mL^−1^ CS, (**f**) GdDOTA acetic acid, 1.5 mg·mL^−1^ CS after 6 and 24 h. Unloaded CS-TPP/HA NGs obtained in citric and acetic acid were used as controls. Scale bar = 50 µm. The green fluorescence intensity were analysed and quantified using the OpenComet program. Magnification, ×100.

**Figure 7:**
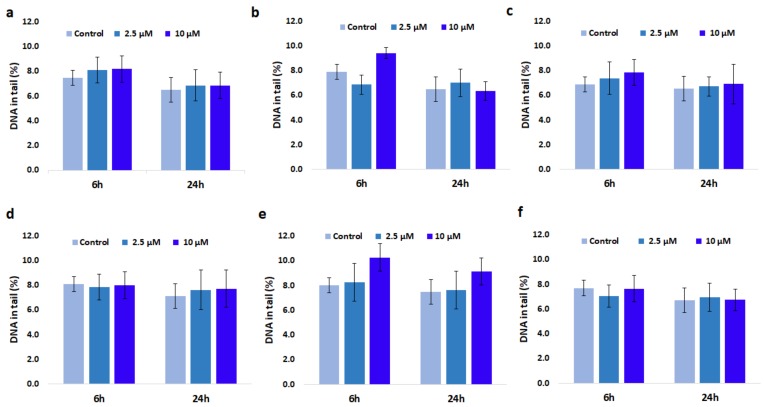
Genotoxicity response of SVEC4-10 cells exposed to different concentrations of (**a**) GdDOTP citric acid, 2.5 mg·mL^−1^ CS; (**b**) GdDOTP acetic acid, 2.5 mg·mL^−1^ CS; (**c**) GdDOTP citric acid, 1.5 mg·mL^−1^ CS; (**d**) GdDOTP acetic acid, 1.5 mg·mL^−1^ CS; (**e**) GdDOTA citric acid, 1.5 mg·mL^−1^ CS; (**f**) GdDOTA acetic acid, 1.5 mg·mL^−1^ CS, after 6 and 24 h. Unloaded CS-TPP/HA NPs obtained in citric and acetic acid were used as controls. Data are expressed as average ± SD (*n* = 3).

In order to check if nanogels can cause damage to nuclear DNA in SVEC4-10 cells, the alkaline Comet assay was performed (Figure 6 and Figure 7). The results showed that nanosuspensions containing 2.5 mg·mL^−1^ CS did not induce formation of DNA strand breaks in SVEC4-10 cells even at higher dose applied for 24 h. Furthermore, no significant difference was observed between cells treated with nanogels in citric phase and those treated with nanogels in acetic phase. In case of cells exposure to nanosuspensions containing 1.5 mg·mL^−1^ CS, slight but significant changes were noticed. Thus, interaction of cells for 6 and 24 h with a higher concentration of citric GdDOTA⊂CS-TPP/HA NGs (e) induced 10.3% and 9.1% DNA in the tail, compared to 8% and 6.5% for unloaded nanogels (CS-TPP/HA NGs). In addition, regardless of nanogels type and the applied dose, after 24 h a downward trend of DNA in tail was observed but without a statistical significance.

## 4. Discussion

The high potential of Gd-based nanogels in developing of new nanocarriers for theranostic applications have made these nanoparticles to be of great scientific interest for biomedical research in the last years. Taking into account that they are designed for in vivo imaging of lymphatic nodes and administrated by subcutaneous injection, biocompatibility of Gd-based nanogels is a critical issue that needs to be considered.

GdCA⊂CS-TPP/HA nanogels (GdCA = GdDOTA or GdDOTP) were prepared by a one-step ionotropic gelation process [61,63]. This method relies upon the establishment of multivalent electrostatic interactions between CS derivatives (polycationic) and HA (polyanionic). The resulting supramolecular network can be reinforced by cross-linking mediated by small anionic cross-linkers such as TPP. For each CS concentration, nanogel mean sizes and their zeta potential were comparable, as well as the polydispersity indexes (PdI) of the corresponding nanosuspensions.

We have recently shown that in order to have a better insight in nanogel mean sizes, DLS data should be systematically consolidated by AFM observations in liquid mode [67]. Indeed, AFM is particularly well suited since this technique requires not only experimental conditions that are not invasive for nanogels but also no application of mathematical models to obtain size information. No topological differences could be noticed according to CS concentration, the acid or the gadolinium contrast agent. In any case, nanogels (NGs) diameters calculated from the AFM images were highly inferior to 100 nm (Table 2) as already demonstrated before [67].

Gd loadings were superior for formulations where CS concentration was 2.5 mg·mL^−1^. This could be interpreted considering that the higher the concentration of CS, the denser the network of the nanogel and the more abundant the CS NH_3_^+^ groups. As a result, electrostatic interactions with anionic gadolinium chelates (GdDOTP^5−^ and GdDOTA^−^ respectively) were promoted, resulting in higher concentrations of contrast agent.

Regarding the biological impact of nanogels on SVEC4-10 murine lymph node endothelial cells, no significant difference in cell survival profiles were noticed in our experiments according to CS concentration, type of acid or gadolinium contrast agent. These results are in agreement with two other cytotoxicity studies regarding exposure of Gd-based nanogels, to C6 glioma cells [61] and fibroblasts from rat skin cells [63].

A previous study on A20 cells viability [66] revealed that, while GdDOTA-NGs had no significant effect on cell survival profiles up to 24 h, GdDOTP-NGs generated a decrease of cell viability in a time- and dose-dependent manner. A20 cell line seemed to be more susceptible to Gd-based nanogels treatment compared with SVEC4-10 endothelial cell line used in the current work. A cell-type-dependence of chitosan cytotoxic effects was reported by Wimardani et al. [71]. Regardless of CS concentration, Gd-based NGs type and exposure time, LDH release in the culture medium also revealed that Gd-loaded hyaluronic acid-chitosan-based nanogels caused no membrane destabilization and had no impact on cell homeostasis, confirmed also by SRB assay results.

In the present work, both cell viability and LDH release studies suggested the safety of these nanogels for SVEC4-10 murine lymph node endothelial cells, that are in accordance with biocompatibility and low-toxicity of CS [21] and HA [43]. Furthermore, our findings are in line with those generated by Baharifar et al., showing CS concentration as the main factor in determining cell viability [72]. Thus, maximum levels of CS concentration applied (2.0 mg·mL^−1^) induced minimum toxicity of nanoparticles in MRC-5 cells.

Because not all defects in membrane or metabolic function are generated by disruptive effects, in order to determine the sub-lethal effects of nanoparticles, more extensive in vitro toxicity studies, such as oxidative stress were necessary [73].

Generation of reactive oxygen species (ROS) leading to oxidative stress was frequently associated with nanoparticles toxicity [74]. The antioxidant system plays a crucial role in maintaining cellular homeostasis. Under normal conditions, cells have the ability to maintain the equilibrium between generation and elimination of ROS. When production of ROS increases or ROS-scavenging capacity decreases, alterations in cellular redox status could occur leading to oxidative stress [75]. The cell antioxidant defence system can regulate the excess of ROS response under physiologic conditions and also protect against toxic agents. This defence system includes antioxidant enzymes and low molecular weight free-radical scavengers. The major non-enzymatic cellular antioxidant is glutathione (GSH), a tripeptide whose antioxidant function is provided by the sulfhydryl group of cysteine.

Previously, we have reported increases in ROS levels in a time dependent manner as well as reductions of GSH concentrations [66]. The present study showed a low and insignificant decrease of GSH level after exposure for 24 h at the highest dose of citric GdDOTA⊂CS-TPP/HA (e) nanogel with 1.5 mg·mL^−1^ CS.

For SVEC4-10 cells treated with GdDOTP⊂CS-TPP/HA (a and b) in both citric and acetic phase with CS concentration of 2.5 mg·mL^−1^ no GSH depletion and ROS generation were induced.

Due to its essential role in cell survival involving the regulatory effect on glutathione synthesis, Nrf-2 transcription factor (NF-E2 p45-related factor) is widely recognized as the master regulator of cellular redox homeostasis [76]. It is well known that, in cells where ROS levels are higher and GSH concentrations are lower, Nrf-2 disruption occurs.

The insignificant changes in Nrf-2 protein activity between Gd-loaded and unloaded-NGs exposed cells noticed during our experiments could be explained by the fact that, due to their antioxidant equipment, endothelial cells have the ability to handle the mild ROS stress generated.

Under oxidative stress, ROS attack the major classes of biological molecules, such as lipids proteins and DNA, leading oxidative damage [77]. Exposure to ROS causes a chain-reaction process in biological systems that induces firstly lipid peroxidation.

As an important marker of lipid peroxidation, a time-dependent increase of MDA levels was observed for nanogels with a CS concentration of 1.5 mg·mL^−1^ compared to those with of 2.5 mg·mL^−1^. The most significant changes of MDA levels by 24%, 20% and 17% after 24 h of exposure to citric GdDOTA⊂CS-TPP/HA (e) and GdDOTP⊂CS-TPP/HA (c) and acetic GdDOTP⊂CS-TPP/HA (d) respectively, relative to unloaded ones, could suggest that cell antioxidant defence system was able to counteract the oxidative stress induced. On the other hand, these results may be at least partly explained by the susceptibility of polyunsaturated fatty acids of lipids to oxidative modification compared to other macromolecules (e.g., nuclear DNA) [75].

Upon cellular stress conditions, multiple genotoxic responses could be generated, one of them being DNA breakage.

Nanosuspensions containing 2.5 mg·mL^−1^ CS did not elicit a genotoxic effect on SVEC4-10 cells at any dose tested and for both genotoxicity endpoints considered. Furthermore, irrespectively to the nanogels types and applied dose, the longer treatment time resulted in a downward trend of DNA strand breaks compared to the 6 h treatment. This could be due to the DNA repair processes, involved in the case of sub-lethal effects in order to ensure survival of cells [77].

On the other hand, the better results for DNA damage than those for lipid peroxidation could be explained by the fact that, due to its double helix structure, nuclear DNA is less vulnerable to oxidative modifications, compared to lipids [75].

Taking into account all these results, Gd-loaded hyaluronic acid-chitosan based nanogels with CS concentration of 2.5 mg·mL^−1^ generated lower oxidative stress compared to those with CS concentration of 1.5 mg·mL^−1^. Probably, this might be due to the chitosan free radical scavenger activity [78] that increases in a concentration-dependent manner [79] and to the protective effect of HA against oxidative stress [80,81].

## 5. Conclusions

In summary, the present study indicates that interaction of Gd-loaded hyaluronic acid-chitosan based nanogels in the dose range of 1–10 µM with SVEC4-10 cells occurred without affecting the viability and membrane integrity, even after 24 h. The cells survival profiles seemed to be independent on the chitosan concentration. As a result of significant ROS generation, a low but insignificantly decrease of GSH concentration and a time-dependent increase of MDA level were produced by citric GdDOTA⊂CS-TPP/HA nanogel with chitosan concentration of 1.5 mg·mL^−1^, especially at the highest dose applied. The insignificant differences in Nrf-2 protein expression between Gd-loaded NGs-exposed cells and the relevant controls suggested that an oxidative stress was slightly induced but this point affected neither redox homeostasis nor cell survival.

Therefore, due to their good biocompatibility with lymph node endothelial cells, Gd-loaded hyaluronic acid-chitosan based nanogels with a concentration in chitosan of 2.5 mg·mL^−1^ appears to be better candidates for future MRI lymph node studies.

## Figures and Tables

**Table 1:** Nanogel characteristics and Gd(III) loadings of GdCA⊂CS-TPP/HA nanogels (NGs) according to chitosan (CS) concentration in the acid (citric or acetic) phase.

**[CS] mg/mL**	**2.5**			
GdCA	GdDOTP		GdDOTA	
Acid	Citric	Acetic	Citric	Acetic
Entries	1	2	[67]	
D_H_ (nm)	242	197	217	393
PdI	0.2	0.4	0.2	0.4
ζ (mV)	31.9	22.7	30.3	31.9
[Gd]_t_ M	2.39 × 10^−4^	7.82 × 10^−4^	9.87 × 10^−5^	9.07 × 10^−4^
**[CS] mg/mL**	**1.5**			
GdCA	GdDOTP		GdDOTA	
Acid	Citric	Acetic	Citric	Acetic
Entries	3	4	5	6
D_H_ (nm)	199	234	186	185
PdI	0.2	0.3	0.2	0.3
ζ (mV)	22.2	18.9	20.4	25.3
[Gd]_t_ M	1.11 × 10^−4^	3.30 × 10^−4^	4.90 × 10^−5^	2.64 × 10^−4^

**Table 2:** Nanogels **1** to **6** diameters (d_AFM_) in function of CS concentration and Gd contrast agent.

Diameters	1	2	3	4	5	6
d_AFM_ ± sd (nm)	69 ± 8	72 ± 7	53 ± 6	55 ± 6	51 ± 10	62 ± 7

CS concentration did not seem to have an impact on the particle diameters.

**Table 3:** The viability of SVEC4-10 cells exposed to unloaded nanogels compared to untreated cells measured by SRB assay. Results are expressed relative to the untreated cells (100%) and represented as average ± SD (*n* = 3).

Time (h)	Control (Untreated Cells)	Unloaded CS-TPP/HA, [CH]_polycation phase_ (mg·mL^−1^)			
		2.5		1.5	
		Citric Acid	Acetic Acid	Citric Acid	Acetic Acid
6	100 ± 2.05	96.34 ± 1.30	100.00 ± 3.70	93.00 ± 1.00	92.00 ± 3.31
24	100 ± 2.4	107.51 ± 6.70	103.55 ± 4.80	98.00 ± 2.70	103.86 ± 1.91
